# Projected Range Contractions of European Protected Oceanic Montane Plant Communities: Focus on Climate Change Impacts Is Essential for Their Future Conservation

**DOI:** 10.1371/journal.pone.0095147

**Published:** 2014-04-21

**Authors:** Rory L. Hodd, David Bourke, Micheline Sheehy Skeffington

**Affiliations:** 1 Plant Ecology Research Unit (PERU), Botany and Plant Science, National University of Ireland Galway, Galway, Ireland; 2 Department of Botany, and Trinity Centre for Biodiversity Research, School of Natural Sciences, Trinity College, Dublin, Dublin, Ireland; Lakehead University, Canada

## Abstract

Global climate is rapidly changing and while many studies have investigated the potential impacts of this on the distribution of montane plant species and communities, few have focused on those with oceanic montane affinities. In Europe, highly sensitive bryophyte species reach their optimum occurrence, highest diversity and abundance in the north-west hyperoceanic regions, while a number of montane vascular plant species occur here at the edge of their range. This study evaluates the potential impact of climate change on the distribution of these species and assesses the implications for EU Habitats Directive-protected oceanic montane plant communities. We applied an ensemble of species distribution modelling techniques, using atlas data of 30 vascular plant and bryophyte species, to calculate range changes under projected future climate change. The future effectiveness of the protected area network to conserve these species was evaluated using gap analysis. We found that the majority of these montane species are projected to lose suitable climate space, primarily at lower altitudes, or that areas of suitable climate will principally shift northwards. In particular, rare oceanic montane bryophytes have poor dispersal capacity and are likely to be especially vulnerable to contractions in their current climate space. Significantly different projected range change responses were found between 1) oceanic montane bryophytes and vascular plants; 2) species belonging to different montane plant communities; 3) species categorised according to different biomes and eastern limit classifications. The inclusion of topographical variables in addition to climate, significantly improved the statistical and spatial performance of models. The current protected area network is projected to become less effective, especially for specialised arctic-montane species, posing a challenge to conserving oceanic montane plant communities. Conservation management plans need significantly greater focus on potential climate change impacts, including models with higher-resolution species distribution and environmental data, to aid these communities' long-term survival.

## Introduction

Mountain regions are thought to be especially susceptible to the effects of climate change [1]. Oceanic mountains, subject to both maritime and continental influences, present a number of difficulties for climatic change predictions and are likely to be significantly different from those of lowland regions, with altitudinal gradients and orographic effects adding to the local spatial and temporal variability [2], [3].

Climate change is projected to have far-reaching impacts on biodiversity and may lead to widespread changes in species distribution and community composition among a variety of taxa throughout the world [4], [5]. Among the many impacts of climate change are the observed and projected changes in species altitudinal [6], [7] and geographical range [8], [9]. Species vulnerable to higher temperatures either contract in range with increasing warmth, to occupy areas of higher altitudes, or move their range northwards, becoming extinct in more southerly regions [5]. Many factors influence a species' ability to alter its range in response to climate change, including dispersal ability and availability of suitable habitat. Species migration is also curtailed by natural barriers, habitat fragmentation and human impacts [10]. Therefore, it is likely that many species will be unable to disperse at rates necessary to keep up with rapidly changing climatic conditions [11]. On the other hand, there is a growing body of phylogenetic evidence to suggest that long-distance dispersal has occurred for a range or arctic-alpine plant species under past changing climates [12], [13], [14], indicating that dispersal may not be as limiting a factor to the future survival of some species as previously thought.

Montane plant species and communities are particularly vulnerable to climate change [15], [16] and have already sustained many alterations in distribution and species composition, including an increased abundance of generalist species, at the expense of specialised arctic-montane and arctic-alpine species [7], [17], [18], [19], [20]. Arctic-montane species belonging to oceanic mountain ranges are particularly adapted both physically and physiologically to grow in harsh, cold conditions [21]. Consequently they have poor competitive and dispersal ability and may encounter difficulties in adjusting rapidly and efficiently to changes affecting their environment [22] and, therefore, have an increased extinction risk [23]. This extinction risk is especially high in oceanic mountain ranges, as vulnerable species in these areas are likely to experience a physiologically-induced decrease in competitive ability in response to climate change [24]. Additionally, the oceanic mountain ranges of Europe tend to be lower than continental ranges, and therefore have no nival zone to accommodate upward migration of vulnerable species [25]. However, relatively few studies have been carried out to date on climate change effects on oceanic and coastal montane vegetation [26] and its responses may be different.

**Figure 1:**
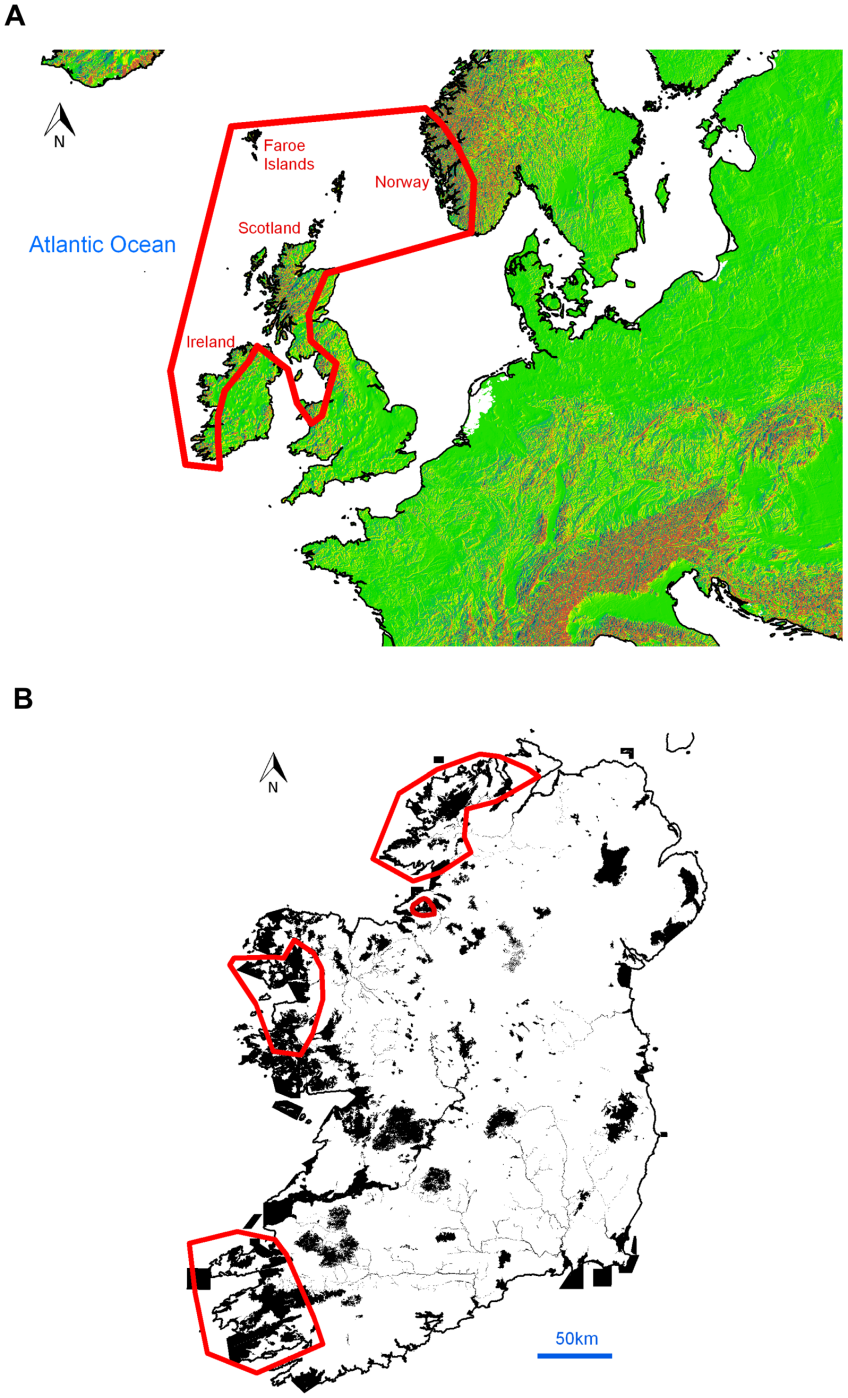
Maps showing the distribution of oceanic montane vegetation in Europe and Ireland. (A) Distribution range (red line) of temperate oceanic montane vegetation in Europe. (B) Main areas (red lines) where conditions are currently optimal for the occurrence of oceanic montane vegetation in Ireland, i.e. areas of altitude over 500 m, that are within the area defined as hyperoceanic by [94]; shaded areas are sites designated as the Natura 2000 network of protected areas (including marine sites) on the island of Ireland.

Bryophytes are ideal for use as predictors and indicators of past, present and future climate change [27], [28], as they often react rapidly to changes in climate [29], [30], [31]. Distributional changes have been detected in a number of British, Irish and European bryophytes, likely as a result of climate change [31], [32], including the northward and eastward shift of south-westerly distributed oceanic bryophytes and an increased vulnerability of northern, montane bryophyte species [32]. Oceanic bryophytes in Europe reach their optimum occurrence and greatest diversity in the hyperoceanic regions of western Scotland and western Ireland (Figure 1A); south-western Ireland has the highest diversity and abundance of Atlantic bryophytes [33]. These bryophyte assemblages, not found elsewhere in Europe, play an essential role locally in community structure and composition [34], [35]. In contrast to widespread lowland bryophytes [36], many oceanic species are particularly vulnerable to changes in climate, as they are not known to produce sporophytes in these areas, spreading only by vegetative means, resulting in slow and unpredictable establishment of new populations [37]. The distribution of these species is also limited by their highly specific microclimatic requirements, which are only met in areas where both climate and local topography are ideal [33], [35].

The montane oceanic species and vegetation of western Europe are of high conservation value at both a national and international scale [34]. They occur in a highly sensitive and vulnerable landscape in upland, temperate regions [38]. As these largely montane heath communities are of such restricted distribution, Ireland and the UK have special responsibility under the EU Habitats Directive to conserve them, particularly those supporting rare assemblages of oceanic bryophytes [34], [39]. These include montane moss heath, dominated by *Racomitrium lanuginosum* and a community of large leafy liverworts, known as ‘mixed northern hepatic mat', which is restricted to shady north-facing mountain slopes in western Scotland and western Ireland [35]. The constituent species of the latter are of high conservation value, as many have a very disjunct global distribution and are highly restricted within Europe, being considered rare or threatened on both an Irish and European scale [39], [40].

Currently, conservation strategies rarely consider climate change [41], [42]. Yet, most plant and vertebrate species have been projected to lose climatically suitable space within protected European conservation areas [42], suggesting that the current protected areas network will be less effective under future climate change, as species ranges may shift to non-protected areas [43], [44], [45]. This will require a range of new conservation approaches and conservation plans that accommodate shifts in species' range by incorporating potential future species ranges [42], [45], [46]. To this aim, knowledge of the potential species' response to future environmental change, including range contractions, will help target limited conservation resources to those species and populations most at risk. Therefore, the projected potential range changes for key montane oceanic heath species in this study will be spatially related to existing protected areas in order to assess the adequacy of these for species conservation under future climate change.

We used a state-of-the-art ensemble species distribution modelling (SDM) approach, which is a conservation planning tool that statistically correlates current species distributions with climatic and other environmental variables and enables projections of future potential distributions. Because of the hepatic mat species' very specific topographical requirements and the restricted occurrence of vascular arctic-montane species in the region, we used topography as a key modelling variable to add precision to the projections. Our aims were to:

- project changes in the future potential distribution of upland bryophyte and vascular plant species under a changing climate and assess the potential implications for oceanic montane plant communities
- investigate the effect of the addition of topographical variables on model predictive capacity for species distribution, since many upland bryophyte and vascular plant species are confined to areas with specific topographical characteristics
- assess the implications of climate change-induced shifts in species range for future conservation management of European protected montane habitats

## Materials and Methods

### Study area

The study area consists of the whole island of Ireland (both the Republic and Northern Ireland), on the north-western fringe of Europe (Figure 1A) between 51°25′N and 55°25′N; 5°26′W and 10°37′W. Ireland has a temperate oceanic climate, with mean monthly temperatures ranging from 6–6.5°C in January to 15–15.5°C in July [47] and relatively high (750 mm to >2500 mm per annum) and frequent (150 to >220 days per annum) rainfall [48]. Rising to 1041 m, most of the mountains are coastal and, in the western part of the country have a hyperoceanic climate, which is highly suitable for the growth of oceanic montane vegetation [34] (Figure 1B). Global climate change is projected to alter the climate of Ireland; temperatures are predicted to rise by 3–4°C by the end of this century, while an increased seasonal variation in rainfall amounts is projected, leading to lower summer and higher winter precipitation [49], [50].

### Species data and selection criteria

The species in this study are either confined to montane habitats or form an important component of montane vegetation. Thirty species (14 bryophyte and 16 vascular plant species) were selected (Table S1), representing four groups: characteristic of, or restricted to, montane heath vegetation; characteristic of montane cliffs; characteristic of mixed northern hepatic mat (‘hepatic mat’) and oceanic bryophyte species that frequently grow in association with hepatic mats, but also occur in a wider range of upland habitats, henceforth referred to as ‘oceanic montane bryophytes’. The species selected for use in these models were either species that were considered to be key components of Irish montane vegetation, based on analysis of ecological data collected from montane vegetation in western Ireland [51], or species that are restricted only to montane vegetation within the study area, selected using a combination of expert knowledge and analysis of the current known distribution of these species [35], [39], [52].

Species were also allocated to specific groups: 1) having a wide, narrow or disjunct distribution; 2) being associated with a particular biogeographical element, divided into biome and eastern limit categories (after [53] for vascular plants and [54] for bryophytes); 3) the maximum and minimum altitude of occurrence (following [52] for vascular plants; [55] for mosses and [56] for liverworts). The eastern limit category is a measure of how far east a species' distribution stretches, using Britain and Ireland as a reference point, ranging from hyperoceanic to circumpolar.

The data for most species were obtained from the National Biodiversity Network (NBN) Gateway [57] and the data for a number of rare oceanic bryophyte species were obtained from the National Parks and Wildlife Service (NPWS), Dublin [58]. These data represent high quality presence-absence distribution data on a 10 km×10 km grid cell basis (Table S2), which is the smallest available grid size for the area studied. No information was available on the abundance of each species within each grid cell, but the majority of species selected form a significant component of the vegetation in which they occur and are likely to occur at multiple locations across each 10 km×10 km grid cell in which they are present.

### Climate data

Climate data from the 1961–1990 baseline period were obtained for the whole island of Ireland [50], [59]. These 10 km×10 km (Irish National Grid) resolution data were derived from daily climate data from 560 precipitation stations and 70 temperature stations spatially interpolated using a polynomial regression method with an inbuilt adjustment for elevation. Variables used include mean, minimum and maximum monthly temperatures, and mean monthly precipitation as well as various derived bioclimatic variables (e.g. net annual rainfall, mean winter temperature, continentality index). The climate change data used in the current study incorporated the mean values of A2 and B2 scenarios obtained from statistically downscaled outputs from the HadCM3 Global Climate Model [59].

### Topographic data

A range of topographical data were extracted from the GTOPO30 digital elevation model (DEM) [60] for each 10 km×10 km grid cell, including mean, maximum and minimum elevation, area of land surface >350 m, area of land surface >500 m, mean slope, area of land surface occupied by aspects facing north-west, north and north-east. Based on the known ecological and topographical conditions required by the species [52], [55], [56], we quantified the area of land surface in each grid cell occupied by those specific topographical variables for inclusion in the models. For example, for the montane heath species we quantified the area of land surface in each grid cell occupied by elevations >350 m, with an aspect facing north-west, north and north-east. Although these species are not restricted exclusively to areas where these conditions are met within the study area, they would very rarely grow at lower altitudes or with other aspects, and reach their optimum occurrence at these aspects and altitudes. Data processing was undertaken in ArcGIS v9.3 (ESRI, Redlands, CA, USA). Hawth's Tools, an extension to ArcGIS, was used to carry out the polygons in polygon analysis [61].

### Variable selection

Sixty climatic and fifteen topographic variables were considered for analysis. All were tested for collinearity, then selected to avoid this, using variance inflation factors (VIFs). These were calculated between the variables and those with the highest VIFs were eliminated until the VIFs for all variables were below a value of 5, sufficiently low to avoid collinearity [62]. Knowledge of the species' ecological requirements informed the final selection of the most ecologically important variables.

The variables used for species of montane heaths and cliffs, were: December minimum temperature, July maximum temperature, mean winter precipitation (December – February), mean slope and area of the land surface in each grid cell >350 m, with an aspect of north to north-east. The variables used for hepatic mat and oceanic bryophyte species were: December minimum temperature, May maximum temperature, mean summer precipitation (June – August), area of cell with slope >10% and area of the land surface in each grid cell >500 m, with an aspect of north to north-east (Table 1).

**Table 1:** Current mean, minimum, maximum and range values for climatic and topographic variables and projected climate variables for 2055.

Climate variables	Units	Used for	Mean	Min	Max	Range
May maximum temperature	°C	Oceanic bryophytes	14.12	12.17	15.09	2.92
July maximum temperature	°C	Montane heath	18.36	15.77	19.63	3.85
December minimum temperature	°C	All species	2.39	−0.57	4.87	5.44
Mean winter precipitation (December-February)	Mm	Montane heath	124.10	63.82	224.99	161.17
Mean summer precipitation (June-August)	Mm	Oceanic bryophytes	83.84	49.64	127.90	78.27

Climate variables – 2055			Mean 2055	Min 2055	Max 2055	Range 2055
May maximum temperature	°C	Oceanic bryophytes	15.75	13.11	15.09	3.43
July maximum temperature	°C	Montane heath	22.61	16.37	19.63	7.85
December minimum temperature	°C	All species	3.48	0.12	4.87	5.81
Mean winter precipitation (December-February)	Mm	Montane heath	137.67	62.34	224.99	181.99
Mean summer precipitation (June-August)	Mm	Oceanic bryophytes	61.10	26.11	127.90	97.77

Topography variables			Mean	Min	Max	Range
Area with aspect northeast to northwest, above 500 m altitude	Km^2^	Oceanic bryophytes	0.14	0.00	20.92	20.92
Area with aspect northeast to northwest, above 350 m altitude	Km^2^	Montane heath	0.46	0.00	24.30	24.30
Area of slope >10%	Km^2^	Oceanic bryophytes	1.66	0.00	40.50	40.50
Mean slope	°	Montane heath	1.18	0.00	5.02	5.02

Which variable is used for oceanic bryophyte, montane heath, cliff, or all species is also indicated.

### Species distribution modelling

We predicted the distribution of the 30 species using an ensemble of species distribution modelling (SDM) techniques appropriate for the presence-absence species data collated, within the BIOMOD2 framework; Generalized Linear Models (GLM), Random Forests (RF), Generalized Boosting Models (GBM), Artificial Neural Networks (ANN), Flexible Discriminant Analysis (FDA) and Generalized Additive Models (GAM) [63], [64]. Models were created using climatic variables only, or climatic with topographic variables, to establish the effects of topography on species distribution and to investigate the effects on predictions of disregarding topography [65]. The predictive performance of these models was measured using Cohen's Kappa statistic (K), True Skill Statistic (TSS), the Area Under the Curve (AUC) of the Receiver Operating Characteristic, sensitivity and specificity scores. Thresholds used to assess model predictive performance are outlined in Table S3. Only those models obtaining AUC, TSS and Kappa scores above 0.8, 0.6 and 0.6, respectively (i.e. with minimum ‘Good’ rating), were used to build the ensemble models. Model evaluation was carried out by splitting the data; 80% for calibration and 20% for validation. All models were calibrated using the R environment software [66] and followed the default settings of BIOMOD2 [64]. The simulated current and future distributions of the species were compared for the models using both climate and topography variables and the % decrease or increase in range for each species calculated. Central to possible range changes of species under changing climate, is the ability of those species to colonise new potentially suitable areas. For vascular plants and bryophytes, this may depend on species' dispersal ability. However, detailed dispersal distances are unavailable for most species; therefore, we examined two extreme scenarios:

1. Unlimited dispersal; where the entire projected future range of the species is taken to be the actual future distribution.

2. Limited (i.e. no) dispersal; where the future distribution results solely from the overlap between current and projected future range of the species.

### Overlap with protected areas

Gap analysis is a protocol for assessing the extent to which valued biodiversity attributes are represented within protected areas [67], [68], and was used here to calculate the overlap between protected areas and species' current and projected potential future distributions. The species studied here belong to habitats mostly protected in Special Areas of Conservation (SAC) designated under the EU Habitats Directive, typically found in blanket and raised bogs, wet, dry, alpine and subalpine heaths, and siliceous and calcareous rocky slopes and scree [69]. The analysis was performed using current gridded presence/absence data, our future projected distributions for the species and the SAC spatial extent data [70] in which the proportion of each grid cell area occupied by SAC was computed. As the species data used were at a coarser resolution to the SAC data, thresholds were devised to match the datasets. Grid cells were considered protected if their proportion of protected area equalled or exceeded 10%. Comparisons of a range of thresholds (≥2%, ≥5%, ≥10%, ≥20%, ≥30%, ≥50%) were made to rule out threshold effects [67]. The analysis was undertaken using the R environment software [66].

### Statistical analyses

Statistically significant differences in model performance statistics, range changes and change in overlap with protected areas for the various species groups were tested using the Wilcoxon signed-rank test and the significance of between-group relationships was calculated using Wilcoxon rank-sum test, as the data were not normally distributed [66].

## Results

### Model performance

**Figure 2:**
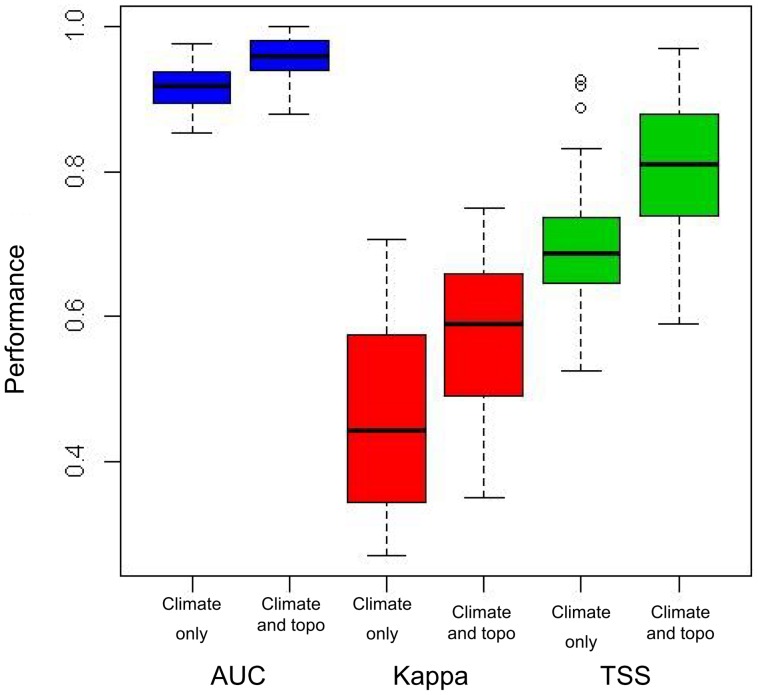
Box plots of the predictive performance of species distribution models for 30 montane species, comparing models with only climate variables to those containing both climate and ecologically relevant topographic variables. The methods of evaluation used were AUC, Kappa and TSS. The difference in performance between models created using climate variables only and climate and topographic variables was significant in all cases (p<0.001).

A comparison of the predictive performance statistics of models using climate variables only and those produced using both climate and topography, revealed that AUC (p<0.01), Kappa (p<0.001) and TSS (p<0.001) were significantly higher for most species, across all modelling techniques, when topography was added to the models (Figure 2; values for individual species in Table S4a). Mean AUC for all species increased from 0.92 (± standard error 0.01) to 0.95 (±0.01); mean Kappa from 0.46 (±0.02) to 0.58 (±0.02) and mean TSS from 0.7 (±0.02) to 0.8 (±0.02) upon addition of topographic variables. Species of disjunct distributions showed a significantly greater increase in performance (p<0.01 for AUC and Kappa; p<0.001 for TSS) than widely distributed species, as did species which are restricted only to montane habitats as opposed to those that are not habitat specific (p<0.01 for all measures of performance). Overall, the models for most species were shown to be very good or excellent at correctly predicting true presences and true absences. Mean (min, max) sensitivity and specificity for all modelled species using both climate and topography were 84.50 (67.40, 100) and 90.25 (76.75, 96.31), respectively (Table S4a). Highly significant correlations were found between all predictive performance statistics, except for Kappa (Table S4b). In general, the models tended to over-predict the current distribution of these species, but the areas identified as suitable were similar to the actual recorded distribution.

### Projected species range changes

**Figure 3:**
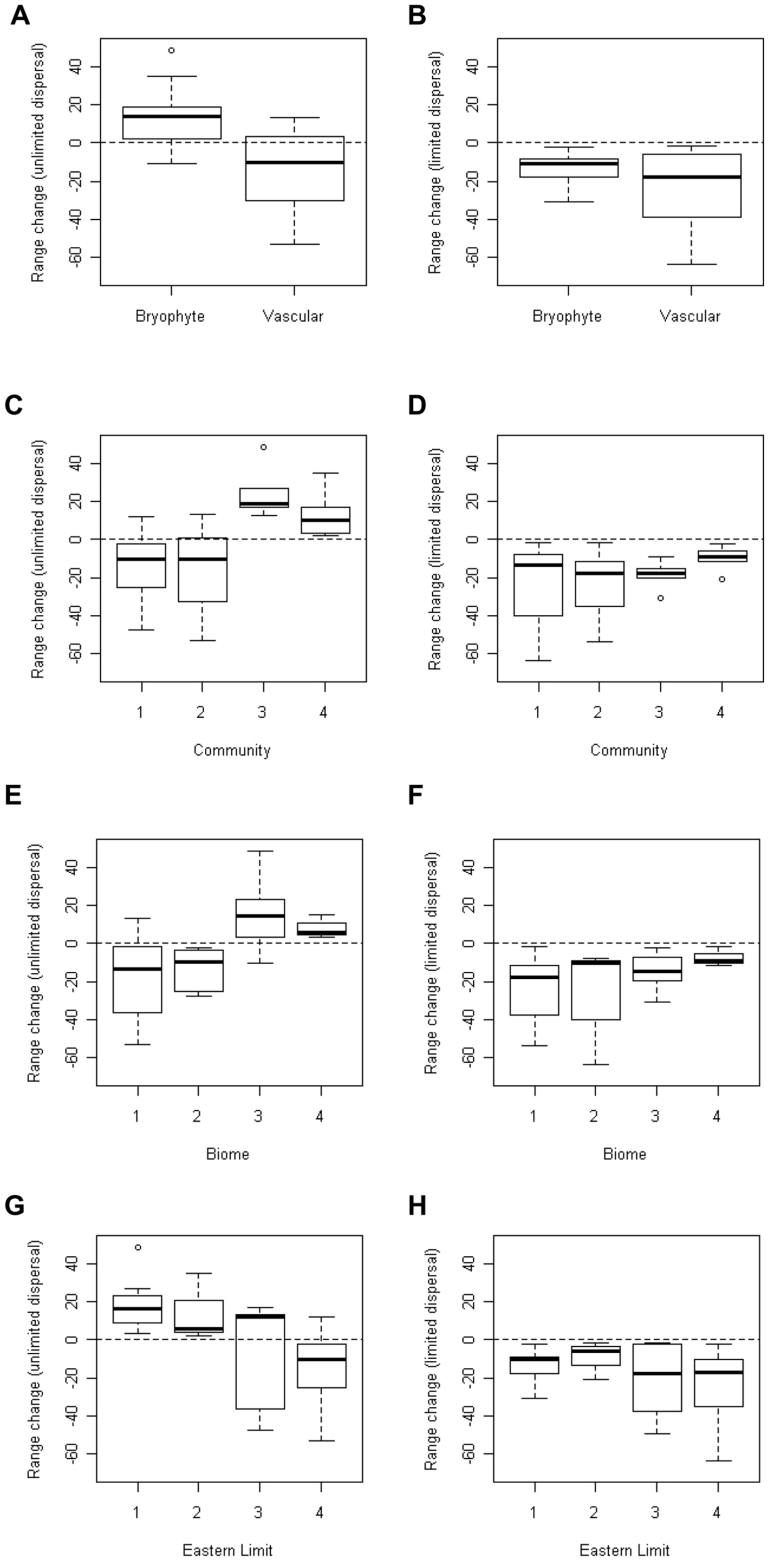
Boxplots of the change in range (%) of species under conditions of unlimited and limited dispersal, grouped by a number of categories. (A)–(B) are grouped by plant type (bryophyte (n = 14) or vascular plant (n = 16)); (C)–(D) are grouped by community (1: montane heath (n = 10), 2: montane cliff (n = 9), 3: hepatic mat (n = 5), 4: oceanic montane bryophyte (n = 6)); (E)–(F) are grouped by biome [53], [54] (1: Arctic-montane (n = 10), 2: Boreo-arctic-montane (n = 5), 3: Boreal montane (n = 12), 4: Temperate and Southern Temperate (n = 3)); (G)–(H) are grouped by eastern limit category [53], [54] (1: hyperoceanic and oceanic (n = 8), 2: suboceanic (n = 3), 3: European, Eurosiberian and Eurasian (n = 5), 4: circumpolar (n = 14)). Range changes calculated using an ensemble of models produced by BIOMOD2.

A variety of range change responses (i.e. changes in areas of suitable climate) were shown for the modelled species under both unlimited and limited dispersal scenarios (Table S5). Comparing bryophyte and vascular plant species under a scenario of unlimited dispersal, they show a significant difference (p<0.001) in their range changes (Figure 3A; means for all categories shown in Figure 3 are listed in Table S6). Bryophytes are projected to show a mean increase in suitable climate space of +12.91% (±4.47), whereas vascular species show a mean decrease of −13.64% (±5.42). Under a limited dispersal scenario (species unable to move to new areas), both bryophyte and vascular plant species decrease in range; bryophytes are projected to show a mean decrease of −12.96% (±2.0), not significantly different from the potential decrease (−23.92%±5.20) of vascular plant species (Figure 3B).

When grouped by community (Figures 3C and 3D), the difference in range change between groups was also significant under unlimited dispersal (p<0.001) but not under conditions of limited dispersal. Only the bryophyte communities (groups 3 and 4), particularly the hepatic mat species (group 4: range change of +25.0% ±6.41, Table S6), show a potential increase in range under unlimited dispersal, but show a notable decrease in range with limited dispersal. In contrast, the species of exposed ridges and montane cliffs (groups 1 & 2) are projected to decrease in range under both unlimited and limited dispersal scenarios, especially under the latter.

When species were grouped by biome and eastern limit category, the differences between the grouping categories were less significant (p<0.01) under an unlimited dispersal scenario and not significant if no dispersal is possible. Under an unlimited dispersal scenario, species within the arctic-montane and boreo-arctic-montane biomes (groups 1 & 2) show a decrease in suitable range (−18.21±7.39 and −13.53±5.37 respectively; Table S6), while the species of the other two biomes show a mean increase (Figure 3E). The range change for the species of the most northerly biomes (1 & 2) is more negative than for the other two groups under a limited dispersal scenario (Figure 3F).

In terms of the eastern limit category, species with circumpolar (group 4; mean change of −13.49%±4.42; Table S6) and easterly (group 3) distributions generally show a mean decrease in range under conditions of unlimited dispersal, while species of suboceanic, oceanic and hyperoceanic distribution show a mean increase in range (Figure 3G); all but one of these are bryophytes. Under conditions of limited dispersal, the range change is broadly similar and negative for all groups (Figure 3H).

**Figure 4:**
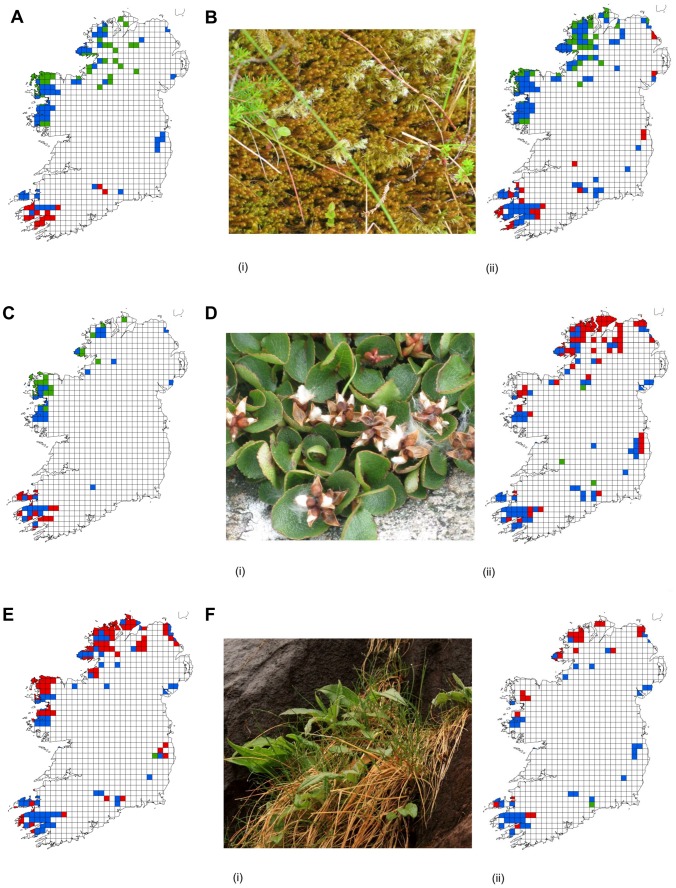
Species distribution maps, showing the projected change in spatial distribution of 6 montane species under predicted climate change scenarios for 2055, representative of the two primary patterns of range change displayed by the modelled species. Species (A) *Anastrepta orcadensis*, (B) *Herbertus aduncus* subsp. *hutchinsiae* ((i) photograph of *H. aduncus* subsp. *hutchinsiae* from Co. Donegal, Ireland; (ii) distribution map) and (C) *Scapania ornithopodioides* are oceanic bryophytes, and show a potential northward shift in range. Species (D) *Salix herbacea* ((i) photograph of *S. herbacea* from Co. Donegal, Ireland; (ii) distribution map), (E) *Sedum rosea* and (F) *Saussurea alpina* ((i) photograph of *S. alpina* from Co. Kerry, Ireland; (ii) distribution map) are of arctic montane distribution, and will potentially contract in range to grid cells of higher altitude. Green grid cells  =  Gain; Blue grid cells  =  Stable; Red grid cells  =  Loss.

A number of potential trends can be identified from the spatially extrapolated model outputs shown as species distribution maps (Figure 4). Oceanic bryophytes (Figure 4A–4C), of hepatic mat and oceanic montane species groups, such as *Anastrepta orcadensis* and *Scapania ornithopodioides*, are projected to lose space to the south and gain space in northerly areas, suggesting a northward shift in distribution of these species (assuming unlimited dispersal). However, species of montane heath and montane cliffs (Figure 4D–4F), many of which are of circumpolar arctic-montane distribution, e.g. *Salix herbacea* and *Sedum rosea*, show a projected general contraction in range to core areas of high altitude, which are centred in the south-west, east and centre-west of Ireland.

### Overlap with protected areas

**Figure 5:**
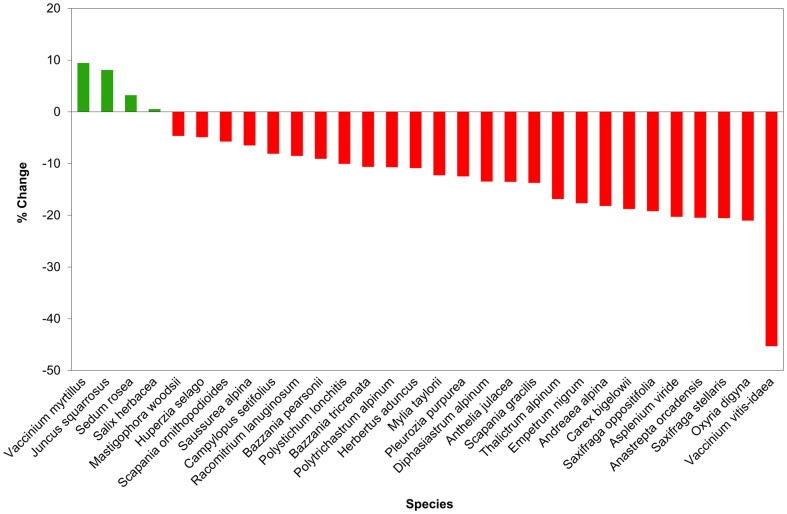
Projected percentage change in overlap with protected areas from current to future distribution, for all species modelled.

**Figure 6:**
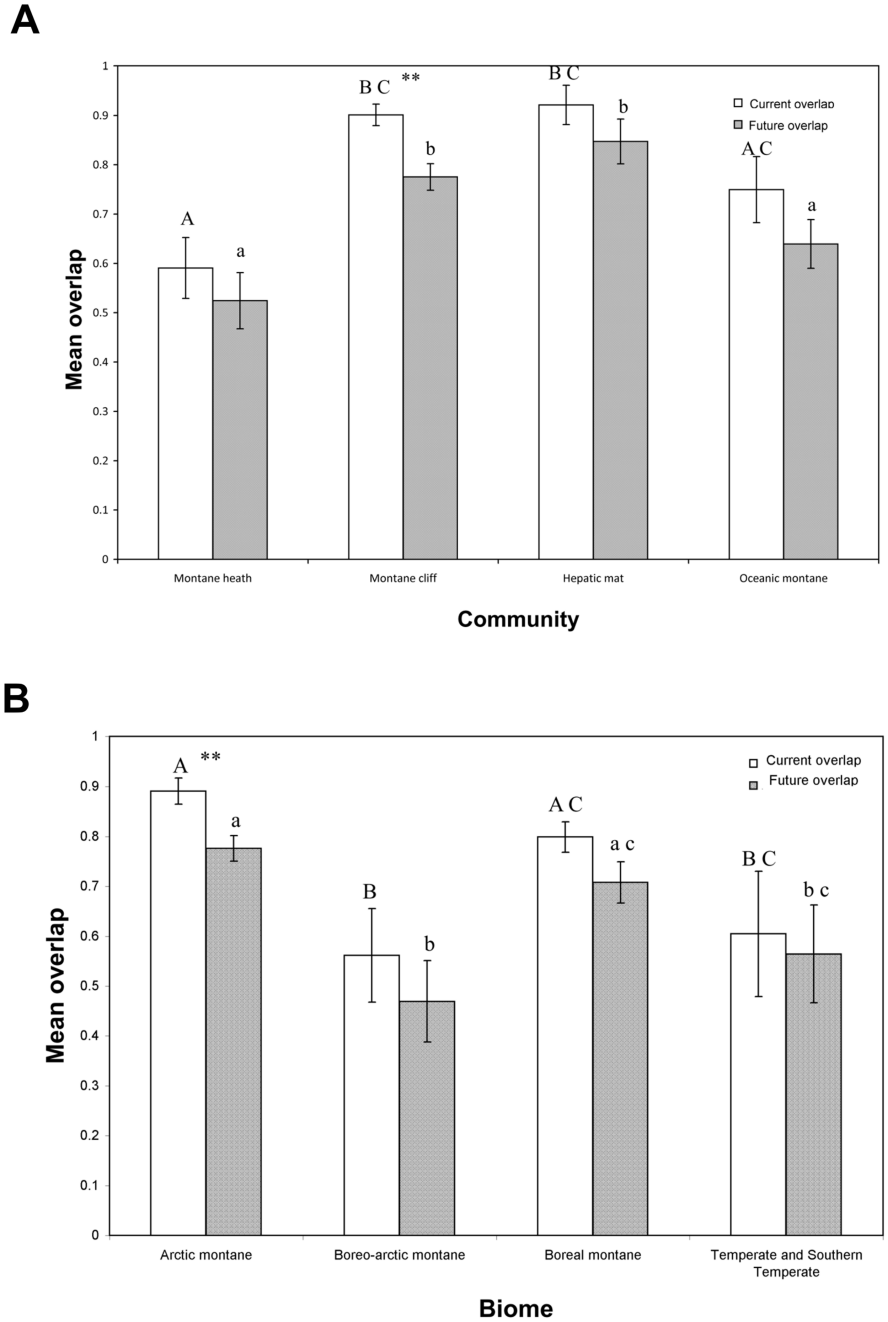
Proportion of overlap between current and future distribution of species groups and protected areas. (A) Grouped by plant community; a different letter signifies a significant difference between categories (Wilcoxon ranked sum; p<0.05; capitals for present, lower case for future scenarios), categories that show a significant change in overlap between current and future (Wilcoxon signed rank; p<0.01) are denoted by **. (B) Grouped by biome; letters signify significant differences between categories (Wilcoxon ranked sum; p<0.05), categories that show a significant change in overlap between current and future (Wilcoxon signed rank; p<0.01) are denoted by **.

The results of the gap analysis indicate that there is projected to be less overlap between the distribution of the majority of species modelled and protected areas in the future (Figure 5; Table S7); in some cases even the current overlap is very low and for some, especially *Vaccinium vitis-idaea*, the future overlap may be extremely low. When grouped by plant community (Figure 6A), the percent overlap with protected areas of species of hepatic mat (92.14%±3.97: current projections; 84.73%±4.53: future projections) and montane cliffs (90.13%±2.19: current projections; 77.53%±2.69: future projections) are significantly (p<0.01) greater than for species of montane heath (59.07%±6.18: current projections; 52.44%±5.68: future projections) under both current and future predictions. Only the distribution of species of montane cliffs (mean decrease of −13.86%±2.73) are projected to show a significant (p<0.01) decrease in overlap with protected areas between the present and future. When grouped by biome (Figure 6B), species of arctic-montane distribution, which under current projections show a high level of overlap with protected areas (89.11%±2.62), are projected to significantly (p<0.01) decrease overlap with protected areas under future climate change scenarios (mean decrease of −12.61%±2.78). Species of Temperate and Southern Temperate affinity show the least projected change in overlap with protected areas.

## Discussion

The results of our study indicate that the montane oceanic vegetation of western Europe is likely to face a number of threats to their future conservation in response to climate change. Arctic-montane species, in particular, are projected to see areas of suitable climate either shift northwards or contract to higher elevations, while internationally important bryophyte species and communities are also under threat. As the effectiveness of the current protected area network is projected to be reduced for these species, climate change may pose a major challenge to the effective conservation of montane oceanic species and plant communities in the future.

### Model performance

This study demonstrates the need to include ecologically relevant topographic variables when projecting species distributions in relation to climate change on a regional scale, especially for species strongly affected by topography and the resultant microclimate. Species distributions are governed by a range of biotic and abiotic factors at different spatial scales [71]. At large continental scales, species distributions are shaped by macroclimate, whereas at smaller local or regional scales, factors such as topography modify this to produce a complex pattern of species distribution and diversity [72]. Species were modelled here at a regional scale and the inclusion of ecologically relevant topographic variables along with climatic variables improved model performance for nearly all species and all modelling techniques. This is important particularly when predicting the distribution of montane plant species [65], while the addition of further autecological information would facilitate the determination of appropriate conservation measures for montane species and habitats [73].

Our study also indicated that the extent of the distribution of a species and the degree to which it is restricted to areas with specific microclimatic conditions influence how well the species model performs when climate variables only are used. Therefore, topographically-dependent oceanic bryophytes, especially hepatic mat species that have a very narrow, often disjunct distribution [33], perform much better when topography is included in the model. Similarly, many arctic-montane species do not model well with only climatic variables, as they are strongly dependent on altitude and other topographic factors to create the required conditions for their growth. The species that exhibit the highest performance statistics when only climate variables are used are generally widely distributed, and also occur outside of montane areas. Therefore, they are generally not restricted by altitude, aspect or other topographic variables and their distribution is more strongly controlled by climatic factors.

The data-splitting cross-validation approach used in our study relies on use of the same data-set, though it has been suggested that this may give an overly optimistic model performance assessment [74]. However, our models were shown to be very good at correctly predicting true presences and true absences, and in the absence of directly relevant independent data-sets and in the knowledge that no predictive modelling will be 100% accurate, this methodology still gives useful results for interpretation [75].

We are fully aware that the relatively coarse-scale resolution for both climate and species distribution data may lead to erroneous predictions, especially in montane regions [76], [77]. Some previous studies have shown greater areas of suitable climate/habitat space predicted using coarser resolution data [71], [77], [78], while others show greater persistence of suitable climate/habitat under finer resolution data [76]. It is possible that by modelling at the 10 km×10 km resolution in the current study we are losing the potential to identify local climate refugia for species persistence and rapid migration [77], particularly in areas such as the west coast of Ireland where there is large local scale variations both in elevation and climate. The averaging of climate variables over broader areas (e.g. 10 km×10 km grids), is likely to result in the loss of the fine-scale variability (e.g. broader ranges of temperature and precipitation values) relevant to the species physiological limitations [77], [78]. However, our knowledge of the species and their ecology has enabled us to infer that the overall projections presented here are entirely probable. While finer-resolution climate and environmental data are available, species data at equivalent resolutions will be an on-going challenge for many regions of the world, including Europe and Ireland. These results, despite their limitations and pending finer-scale data, are important to publicise, since one purpose of this paper is to influence policy-makers in advance of any major range changes occurring.

### Range Changes

This study shows how bryophyte and vascular plant species may respond differently to climate change and oceanic bryophytes may retain a higher proportion of suitable climate space than montane vascular plant species. However, the majority of the oceanic bryophytes are projected to lose a large proportion of their climatically suitable area in the south of their range and gain this in the north, so would only be able to migrate successfully if there were no barriers to their dispersal. It is also possible that some species may adapt *in situ* and therefore may not need to migrate for the species to survive [12], [14]. In contrast, many of the vascular plants of montane heaths and cliff ledges are projected to lose space in areas of lower altitude and become restricted to the areas of highest altitude, with little or no gain of potential climate space. Most species that showed this contraction in range are of arctic-montane distribution, while species of temperate and southern distribution generally showed a projected increase, or minor decrease, in suitable space. Both of these patterns are likely to be in response to temperature changes.

These patterns are also likely to be influenced by rainfall changes, particularly for the widespread oceanic and hepatic mat bryophytes. These results suggest that the degree of oceanicity of a species' distribution is likely to influence its response to climate change. Species of hyperoceanic, oceanic and suboceanic distribution mainly show a pattern of northward shift in range. The specific cause of these shifts in range is not clear.

When considering species' potential range changes, it is essential to take dispersal capacity into account [79]. Neither dispersal scenario used in this study is likely to reflect the species' true dispersal capacity [80], as they are unlikely to be able to colonise every area that may become suitable for their growth in the future, but may shift their range to some extent in response to climate changes. The limited dispersal scenario is likely to be closer to their true response, as most species studied have poor dispersal capacity, are of disjunct distribution and are limited to relatively isolated mountain ranges, creating a further barrier to their dispersal. However, little is known about their true dispersal capacity, particularly of the bryophytes. Liverworts of the hepatic mat community are not known to produce sporophytes in Ireland, spreading only vegetatively, primarily by fragmentation [35].

Biotic interactions are likely to have a major influence on future composition of montane vegetation in the face of climate change. Temperature rises are likely to lead to increased competition in montane habitats [7], [20], with less cold-tolerant species expanding their niche upward in altitude and others, already present in these habitats, growing more efficiently to outcompete specialised montane species [24]. This indirect effect of temperature rises may have a greater negative impact on the range of montane species than other consequences of climate change [81], and may lead to a homogenisation of montane vegetation, as has been detected in Scotland [17]. However, competition may not be as great in exposed montane heath, as exposure and wind strength may limit the growth of many species, negating the impact of rising temperatures, and enabling the survival of adapted arctic-montane species [82]. In contrast, oceanic bryophytes of sheltered habitats, particularly those of the mixed northern hepatic mat, may be more threatened by competition. A combination of decreased rainfall at key periods of the year and temperature rises will likely favour the growth of large acrocarpous and pleurocarpous mosses and vascular plant species, to the possible exclusion of hepatic mat liverworts [35].

Despite the fact that climate change might be too rapid for vulnerable species to migrate to new regions of suitable climate space [83] and may cause a loss in genetic diversity in northern species [84], plants living in marginal areas, such as mountain-tops, have an inherent genetic variability and adaptability at the metapopulation level that may impose a greater degree of resilience on the component species than in more stable habitats [85]. Indeed, arctic-alpine vascular plants can be extremely long-lived [86] and, with some recruitment and genetic variability, species have survived many climatic oscillations [87]. Little is yet known of bryophyte genetic variability [88]. However, when addressing conservation aims in the face of climate change, all potential range changes must be considered and the extension of protected areas examined, as a basic safety-net method to prevent possible regional species extinctions.

### Conservation of montane vegetation in response to climate change

Hyperoceanic plant communities are highly restricted in Europe to the western fringes of Ireland, Britain, Norway and the Faroes; those communities dominated by bryophytes exhibit an even more restricted distribution [24], [89]. However, there is little specific protection of these bryophyte-dominated communities at a European level. As Ireland is at the south-western limit of the range of occurrence of many of these communities and their constituent species, climate change poses a greater threat to their future survival than in other parts of their range. Therefore, Ireland has a major role to play in conserving the European populations of these species. If their range were to move northwards, it would also increase the role of Scotland, Norway and the Faroe Islands in the conservation of this vegetation (*sensu* [90]).

These results suggest there will be a reduction in area of all true montane habitats in Ireland, as defined by their characteristic species. Many are listed under Annex 1 of the EU Habitats Directive [91], including Alpine and boreal heaths (4060), siliceous Alpine and boreal grassland (6150), as well as significant elements of siliceous rocky slope vegetation (8220), calcareous rocky slope vegetation (8210) and hydrophilous tall herb vegetation (6430). Other Annex habitats, currently integral parts of montane vegetation in European oceanic areas, including wet heath (4010), dry heath (4030) and active blanket bog (*7130), may also come under considerable threat with climate change.

A number of the modelled species, as well as others of these habitats that could not be modelled, as they occur in too few grid cells, are listed in the Irish Red Data Books [40], [92], [93] and are already highly threatened in Ireland. It is likely that the further threat of climate change may preclude the future survival of these species.

The results of the gap analysis indicate that the current protected area network in Ireland may not adequately conserve many montane species under future climate change, particularly arctic-montane and boreo-arctic montane species, which are likely to be the most vulnerable species to climate change. Although the modelled oceanic bryophyte species are also projected to lose climate space in protected areas, a large proportion of their range still overlaps with protected areas, in contrast to species of montane heath. This is likely to be due to the fact that the distribution of the oceanic species, by definition, is restricted to western, coastal areas, which is where Irish protected areas are concentrated [34]. These results can inform future strategies for managing and designating protected areas in montane oceanic areas.

## Conclusions

The marginal position of European oceanic regions results in their indigenous species and plant communities being of high conservation value, with a richness and diversity of oceanic bryophytes not seen elsewhere in Europe. The threat to their conservation from climate change is particularly high. The relatively small extent and low altitude of Ireland's mountain areas means that the constituent species of these communities have very little chance of shifting their range to areas of suitable climate in response to climate change. The challenge facing policy makers and conservation organisations is to recognise the uniqueness and value of oceanic vegetation and ensure the highest conservation status of this vegetation in response to the effects of climate change and other threats. By incorporating the results of studies of species distributional changes under future climate change, the robustness and relevance of protected areas and conservation strategies can be ensured into the future.

## Supporting Information

Table S1 **List of species modelled and their main attributes (threat status for bryophytes after** [40] **; threat status for vascular species after** [93] **); biome, eastern limit category and altitude range after** [53] **and** [54] **).**
(DOC)

Table S2 **Presence/absence data for all species for all Irish 10 km x 10 km grid cells.** Data obtained from National Biodiversity Network Gateway [57] and National Parks and Wildlife Service, Dublin [58].
(XLS)

Table S3 **Threshold values for classifying model predictive accuracy** [95] **,** [96] **, where AUC is Area Under the Curve and Kappa/TSS is Cohen's Kappa statistic (K) and True Skill Statistic (TSS) respectively.**
(DOC)

Table S4 A: AUC, Kappa and TSS values for all species using an ensemble modelling approach in BIOMOD2, from models created using both climate and topography variables and climate variables only. Model predictive accuracy is acceptable when AUC>0.7 and Kappa/TSS>0.4 (Table S3). Sensitivity and specificity presented only for models created using both climatic and topographic variables. B: Correlation coefficients between the five model predictive performance statistics: Cohen's Kappa statistic (K), True Skill Statistic (TSS), Area Under the Curve (AUC) of the Receiver Operating Characteristic, Sensitivity and Specificity scores.
(DOC)

Table S5 **Range changes (%), derived from ensemble of models produced in BIOMOD2, for all species under conditions of unlimited and limited dispersal.**
(DOC)

Table S6 **Mean range changes (%) ± standard error of the mean, under conditions of unlimited and limited dispersal, under the categories of plant type, community, biome and eastern limit.**
(DOC)

Table S7 **Outputs from gap analysis, showing the proportional overlap of both current and future distribution of all species with protected areas, using a range of threshold values (≥2%, ≥5%, ≥10%, ≥20%, ≥30%, ≥50% of grid cell occupied by protected areas).**
(DOC)
