# Supplementary material for: Projected Range Contractions of European Protected Oceanic Montane Plant Communities: Focus on Climate Change Impacts Is Essential for Their Future Conservation
Source: PLoS One. 2014 Apr 21;9(4):e95147. doi: 10.1371/journal.pone.0095147 (PMC3994024; doi:10.1371/journal.pone.0095147)
Supplement: Table S1 — List of species modelled and their main attributes (threat status for bryophytes after [40] ; threat status for vascular species after [93] ); biome, eastern limit category and altitude range after [53] and [54] ). (DOC) [file pone.0095147.s001.doc]

Table S1: List of species modelled and their main attributes (threat status for bryophytes after [40]; threat status for vascular species after [93]); biome, eastern limit category and altitude range after [53] and [54]).

| **Species Name** | **Bryophyte/ Vascular plant** | **Threat status in Ireland** | **Threat status in Europe** | **Distribution** | **Restricted to montane habitats** | **Community** | **Biome** | **Eastern limit** | **Altitude range in Ireland and UK (m)** |
| --- | --- | --- | --- | --- | --- | --- | --- | --- | --- |
| *Anastrepta orcadensis* | Bryophyte | Least concern | - | Disjunct | Yes | Oceanic montane | Boreal montane | Suboceanic | 60-1300 |
| *Andreaea alpina* | Bryophyte | Least concern | - | Disjunct | Yes | Oceanic montane | Boreal montane | Oceanic | 0-1170 |
| *Anthelia julacea* | Bryophyte | Least concern | - | Disjunct | Yes | Montane cliff | Arctic montane | Circumpolar | 0-1340 |
| *Asplenium viride* | Vascular plant | Least concern | - | Disjunct | Yes | Montane cliff | Boreal montane | Circumpolar | 0-975 |
| *Bazzania pearsonii* | Bryophyte | Vulnerable | Rare | Disjunct | Yes | Hepatic mat | Boreal montane | Oceanic | 300-1000 |
| *Bazzania tricrenata* | Bryophyte | Least concern | - | Disjunct | Yes | Hepatic mat | Boreal montane | European | 0-1220 |
| *Campylopus setifolius* | Bryophyte | Least concern | Rare | Disjunct | No | Oceanic montane | Temperate | Hyperoceanic | 0-800 |
| *Carex bigelowii* | Vascular plant | Least concern | - | Disjunct | Yes | Montane heath | Arctic montane | Circumpolar | 15-1305 |
| *Diphasiastrum alpinum* | Vascular plant | Least concern | - | Disjunct | Yes | Montane heath | Arctic montane | Circumpolar | 0-1220 |
| *Empetrum nigrum* | Vascular plant | Least concern | - | Wide | No | Montane heath | Boreo-arctic montane | Circumpolar | 0-1250 |
| *Herbertus aduncus* | Bryophyte | Least concern | - | Disjunct | Yes | Hepatic mat | Boreal montane | Oceanic | 0-1040 |
| *Huperzia selago* | Vascular plant | Least concern | - | Wide | No | Montane heath | Boreo-arctic montane | Circumpolar | 0-1310 |
| *Juncus squarrosus* | Vascular plant | Least concern | - | Wide | No | Montane heath | Temperate | Suboceanic | 0-1040 |
| *Mastigophora woodsii* | Bryophyte | Near Threatened | Rare | Disjunct | Yes | Hepatic mat | Boreal montane | Oceanic | 300-1000 |
| *Mylia taylorii* | Bryophyte | Least concern | - | Disjunct | No | Oceanic montane | Boreal montane | Suboceanic | 0-1226 |
| *Oxyria digyna* | Vascular plant | Least concern | - | Disjunct | Yes | Montane cliff | Arctic montane | Circumpolar | 150-1240 |
| *Pleurozia purpurea* | Bryophyte | Least concern | - | Narrow | No | Oceanic montane | Boreal montane | Oceanic | 0-915 |
| *Polystichum lonchitis* | Vascular plant | Rare | - | Disjunct | Yes | Montane cliff | Boreal montane | Circumpolar | 600-1065 |
| *Polytrichastrum alpinum* | Bryophyte | Least concern | - | Disjunct | Yes | Montane heath | Boreo-arctic montane | Circumpolar | 0-1335 |
| *Racomitrium lanuginosum* | Bryophyte | Least concern | - | Wide | No | Montane heath | Boreo-arctic montane | Circumpolar | 0-1340 |
| *Salix herbacea* | Vascular plant | Least concern | - | Disjunct | Yes | Montane heath | Arctic montane | European | 600-1310 |
| *Saussurea alpina* | Vascular plant | Rare | - | Disjunct | Yes | Montane cliff | Arctic montane | Eurasian | 0-1207 |
| *Saxifraga oppositifolia* | Vascular plant | Rare | - | Disjunct | Yes | Montane cliff | Arctic montane | Circumpolar | 300-1211 |
| *Saxifraga stellaris* | Vascular plant | Least concern | - | Disjunct | Yes | Montane cliff | Arctic montane | European | 200-1340 |
| *Scapania gracilis* | Bryophyte | Least concern | - | Narrow | No | Oceanic montane | Southern temperate | Hyperoceanic | 0-850 |
| *Scapania ornithopodioides* | Bryophyte | Vulnerable | - | Disjunct | Yes | Hepatic mat | Boreal montane | Oceanic | 230-1000 |
| *Sedum rosea* | Vascular plant | Least concern | - | Disjunct | Yes | Montane cliff | Arctic montane | Circumpolar | 300-1166 |
| *Thalictrum alpinum* | Vascular plant | Least concern | - | Disjunct | Yes | Montane cliff | Arctic montane | Circumpolar | 300-1209 |
| *Vaccinium myrtillus* | Vascular plant | Least concern | - | Wide | No | Montane heath | Boreal montane | Eurosiberian | 0-1300 |
| *Vaccinium vitis-idaea* | Vascular plant | Least concern | - | Disjunct | No | Montane heath | Boreo-arctic montane | Circumpolar | 30-1095 |
