# Supplementary material for: Projected Range Contractions of European Protected Oceanic Montane Plant Communities: Focus on Climate Change Impacts Is Essential for Their Future Conservation
Source: PLoS One. 2014 Apr 21;9(4):e95147. doi: 10.1371/journal.pone.0095147 (PMC3994024; doi:10.1371/journal.pone.0095147)
Supplement: Table S3 — Threshold values for classifying model predictive accuracy [95] , [96] , where AUC is Area Under the Curve and Kappa/TSS is Cohen's Kappa statistic (K) and True Skill Statistic (TSS) respectively. (DOC) [file pone.0095147.s003.doc]

Table S3: Threshold values for classifying model predictive accuracy [95], [96], where AUC is Area Under the Curve and Kappa/TSS is Cohen’s Kappa statistic (K) and True Skill Statistic (TSS) respectively.

| **Accuracy** | **AUC** | **Kappa/TSS** |
| --- | --- | --- |
| Excellent or high | 0.9 – 1 | 0.8 – 1 |
| Good | 0.8 – 0.9 | 0.6 – 0.8 |
| Fair | 0.7 – 0.8 | 0.4 – 0.6 |
| Poor | 0.6 – 0.7 | 0.2 – 0.4 |
| Fail or null | 0.5 – 0.6 | 0.0 – 0.2 |
