# Supplementary material for: Projected Range Contractions of European Protected Oceanic Montane Plant Communities: Focus on Climate Change Impacts Is Essential for Their Future Conservation
Source: PLoS One. 2014 Apr 21;9(4):e95147. doi: 10.1371/journal.pone.0095147 (PMC3994024; doi:10.1371/journal.pone.0095147)
Supplement: Table S4 — A: AUC, Kappa and TSS values for all species using an ensemble modelling approach in BIOMOD2, from models created using both climate and topography variables and climate variables only. Model predictive accuracy is acceptable when AUC>0.7 and Kappa/TSS>0.4 (Table S3). Sensitivity and specificity presented only for models created using both climatic and topographic variables. B: Correlation coefficients between the five model predictive performance statistics: Cohen's Kappa statistic (K), True Skill Statistic (TSS), Area Under the Curve (AUC) of the Receiver Operating Characteristic, Sensitivity and Specificity scores. (DOC) [file pone.0095147.s004.doc]

Table S4a: AUC, Kappa and TSS values for all species using an ensemble modelling approach in BIOMOD2, from models created using both climate and topography variables and climate variables only. Model predictive accuracy is acceptable when AUC > 0.7 and Kappa/TSS > 0.4 (Table S2). Sensitivity and specificity presented only for models created using both climatic and topographic variables.

|  | AUC | | Kappa | | TSS | | Sensitivity | Specificity |
| --- | --- | --- | --- | --- | --- | --- | --- | --- |
|  | Climate only | Climate and topography | Climate only | Climate and topography | Climate only | Climate and topography | Climate and topography | Climate and topography |
| *Anastrepta orcadensis* | 0.940 | 0.978 | 0.328 | 0.506 | 0.821 | 0.894 | 92.42 | 92.95 |
| *Andreaea alpina* | 0.905 | 0.958 | 0.273 | 0.512 | 0.675 | 0.794 | 80.81 | 91.90 |
| *Anthelia julacea* | 0.929 | 0.979 | 0.459 | 0.726 | 0.715 | 0.877 | 89.47 | 93.33 |
| *Asplenium viride* | 0.899 | 0.939 | 0.378 | 0.473 | 0.658 | 0.805 | 81.40 | 87.25 |
| *Bazzania pearsonii* | 0.977 | 0.994 | 0.343 | 0.480 | 0.926 | 0.969 | 100.00 | 96.31 |
| *Bazzania tricrenata* | 0.928 | 0.958 | 0.518 | 0.633 | 0.724 | 0.790 | 83.33 | 88.58 |
| *Campylopus setifolius* | 0.914 | 0.959 | 0.400 | 0.569 | 0.682 | 0.808 | 81.88 | 93.13 |
| *Carex bigelowii* | 0.882 | 0.970 | 0.324 | 0.643 | 0.632 | 0.834 | 84.13 | 93.67 |
| *Diphasiastrum alpinum* | 0.882 | 0.950 | 0.440 | 0.605 | 0.660 | 0.748 | 81.88 | 88.48 |
| *Empetrum nigrum* | 0.903 | 0.927 | 0.661 | 0.698 | 0.643 | 0.671 | 80.47 | 82.57 |
| *Herbertus aduncus* | 0.945 | 0.973 | 0.557 | 0.706 | 0.789 | 0.826 | 86.21 | 91.64 |
| *Huperzia selago* | 0.864 | 0.891 | 0.575 | 0.664 | 0.563 | 0.609 | 67.40 | 89.98 |
| *Juncus squarrosus* | 0.929 | 0.950 | 0.706 | 0.741 | 0.703 | 0.739 | 77.64 | 93.39 |
| *Mastigophora woodsii* | 0.973 | 0.997 | 0.307 | 0.421 | 0.919 | 0.964 | 100.00 | 95.72 |
| *Mylia taylorii* | 0.854 | 0.879 | 0.444 | 0.540 | 0.526 | 0.616 | 71.70 | 85.73 |
| *Oxyria digyna* | 0.934 | 0.968 | 0.344 | 0.463 | 0.736 | 0.835 | 87.88 | 93.16 |
| *Pleurozia purpurea* | 0.896 | 0.891 | 0.605 | 0.594 | 0.626 | 0.599 | 69.87 | 88.02 |
| *Polystichum lonchitis* | 0.893 | 0.951 | 0.416 | 0.475 | 0.645 | 0.800 | 82.22 | 89.43 |
| *Polytrichum alpinum* | 0.895 | 0.940 | 0.270 | 0.349 | 0.662 | 0.768 | 78.95 | 91.74 |
| *Racomitrium lanuginosum* | 0.899 | 0.920 | 0.619 | 0.688 | 0.658 | 0.672 | 80.69 | 81.78 |
| *Salix herbacea* | 0.911 | 0.967 | 0.443 | 0.639 | 0.696 | 0.854 | 90.10 | 94.22 |
| *Saussurea alpina* | 0.929 | 0.985 | 0.309 | 0.507 | 0.718 | 0.909 | 95.00 | 93.71 |
| *Saxifraga oppositifolia* | 0.940 | 0.969 | 0.302 | 0.492 | 0.831 | 0.876 | 87.88 | 91.00 |
| *Saxifraga stellaris* | 0.938 | 0.989 | 0.587 | 0.749 | 0.736 | 0.935 | 88.70 | 94.54 |
| *Scapania gracilis* | 0.888 | 0.894 | 0.587 | 0.588 | 0.621 | 0.607 | 76.95 | 78.17 |
| *Scapania ornithopodioides* | 0.972 | 0.989 | 0.384 | 0.478 | 0.889 | 0.945 | 96.30 | 95.45 |
| *Sedum rosea* | 0.922 | 0.958 | 0.543 | 0.672 | 0.691 | 0.803 | 89.50 | 88.54 |
| *Thalictrum alpinum* | 0.927 | 0.985 | 0.466 | 0.504 | 0.682 | 0.934 | 91.30 | 94.82 |
| *Vaccinium myrtillus* | 0.939 | 0.964 | 0.587 | 0.629 | 0.749 | 0.855 | 81.44 | 91.62 |
| *Vaccinium vitis-idaea* | 0.857 | 0.897 | 0.541 | 0.636 | 0.534 | 0.591 | 79.62 | 76.75 |

Table S4b: Correlation coefficients between the five model predictive performance statistics: Cohen’s Kappa statistic (K), True Skill Statistic (TSS), Area Under the Curve (AUC) of the Receiver Operating Characteristic, Sensitivity and Specificity scores.

|  | AUC | Kappa | TSS | Sensitivity | Specificity |
| --- | --- | --- | --- | --- | --- |
| AUC | 1 |  |  |  |  |
| Kappa | -0.182^n.s^. | 1 |  |  |  |
| TSS | 0.971*** | -0.305 ^n.s^. | 1 |  |  |
| Sensitivity | 0.882*** | -0.286 ^n.s^. | 0.890*** | 1 |  |
| Specificity | 0.798*** | -0.248 ^n.s^. | 0.821*** | 0.578** | 1 |

n.s. = non-significant

** = p < 0.001

*** = p < 0.0001
