# Supplementary material for: Projected Range Contractions of European Protected Oceanic Montane Plant Communities: Focus on Climate Change Impacts Is Essential for Their Future Conservation
Source: PLoS One. 2014 Apr 21;9(4):e95147. doi: 10.1371/journal.pone.0095147 (PMC3994024; doi:10.1371/journal.pone.0095147)
Supplement: Table S5 — Range changes (%), derived from ensemble of models produced in BIOMOD2, for all species under conditions of unlimited and limited dispersal. (DOC) [file pone.0095147.s005.doc]

Table S5: Range changes (%), derived from ensemble of models produced in BIOMOD2, for all species under conditions of unlimited and limited dispersal.

| **Species Name** | **Range change - Unlimited dispersal** | **Range change - Limited dispersal** |
| --- | --- | --- |
| *Anastrepta orcadensis* | 35.06 | -20.78 |
| *Andreaea alpina* | 5.00 | -2.50 |
| *Anthelia julacea* | -10.68 | -11.65 |
| *Asplenium viride* | 1.20 | -14.37 |
| *Bazzania pearsonii* | 48.84 | -9.30 |
| *Bazzania tricrenata* | 16.78 | -17.48 |
| *Campylopus setifolius* | 15.44 | -11.76 |
| *Carex bigelowii* | -15.97 | -17.65 |
| *Diphasiastrum alpinum* | -10.26 | -16.67 |
| *Empetrum nigrum* | -25.08 | -40.34 |
| *Herbertus aduncus* | 12.88 | -20.45 |
| *Huperzia selago* | -3.26 | -9.20 |
| *Juncus squarrosus* | 5.93 | -1.48 |
| *Mastigophora woodsii* | 27.27 | -15.15 |
| *Mylia taylorii* | 2.21 | -5.90 |
| *Oxyria digyna* | 12.00 | -2.00 |
| *Pleurozia purpurea* | 17.31 | -8.48 |
| *Polystichum lonchitis* | -10.40 | -19.20 |
| *Polytrichum alpinum* | -9.72 | -10.42 |
| *Racomitrium lanuginosum* | -2.14 | -7.65 |
| *Salix herbacea* | -47.41 | -49.63 |
| *Saussurea alpina* | -36.11 | -37.50 |
| *Saxifraga oppositifolia* | -32.41 | -35.19 |
| *Saxifraga stellaris* | 13.39 | -1.57 |
| *Scapania gracilis* | 3.25 | -9.09 |
| *Scapania ornithopodioides* | 19.23 | -30.77 |
| *Sedum rosea* | -53.16 | -53.80 |
| *Thalictrum alpinum* | -1.47 | -17.65 |
| *Vaccinium myrtillus* | 12.26 | -2.42 |
| *Vaccinium vitis-idaea* | -27.44 | -64.02 |
