# Supplementary material for: Projected Range Contractions of European Protected Oceanic Montane Plant Communities: Focus on Climate Change Impacts Is Essential for Their Future Conservation
Source: PLoS One. 2014 Apr 21;9(4):e95147. doi: 10.1371/journal.pone.0095147 (PMC3994024; doi:10.1371/journal.pone.0095147)
Supplement: Table S6 — Mean range changes (%) ± standard error of the mean, under conditions of unlimited and limited dispersal, under the categories of plant type, community, biome and eastern limit. (DOC) [file pone.0095147.s006.doc]

Table S6: Mean range changes (%) ± standard error of the mean, under conditions of unlimited and limited dispersal, for each of the major categories of plant type, community, biome and eastern limit.

|  | **Number of species** | **Range change – Unlimited dispersal** | **Range change - Limited dispersal** |
| --- | --- | --- | --- |
| Vascular plant | 16 | -13.64 ±5.42 | -23.92 ±5.20 |
| Bryophyte | 14 | 12.91 ±4.47 | -12.96 ±2.00 |
|  |  |  |  |
| **Community** |  |  |  |
| Montane heath | 10 | -12.31 ±5.55 | -21.95 ±6.85 |
| Montane cliff | 9 | -13.07 ±7.63 | -21.44 ±5.81 |
| Oceanic montane bryophytes | 6 | 13.05 ±5.12 | -9.75 ±2.55 |
| Hepatic mats | 5 | 25.00 ±6.41 | -18.63 ±3.54 |
|  |  |  |  |
| **Biome** |  |  |  |
| Arctic-montane | 10 | -18.21 ±7.39 | -24.33 ±5.89 |
| Boreo-Arctic-montane | 5 | -13.53 ±5.37 | -26.32 ±11.21 |
| Boreal-montane | 12 | 15.64 ±4.63 | -13.90 ±2.45 |
| Temperate | 2 | 10.69 ±4.75 | -6.62 ±5.14 |
| Southern temperate | 1 | 0.37 | -14.13 |
|  |  |  |  |
| **Eastern Limit category** |  |  |  |
| Hyperoceanic | 2 | 9.34 ±6.10 | -10.43 ±1.34 |
| Oceanic | 6 | 21.76 ±6.19 | -14.44 ±4.11 |
| Suboceanic | 3 | 14.40 ±10.39 | -9.39 ±5.84 |
| European | 3 | -5.75 ±20.85 | -22.90 ±14.13 |
| Eurosiberian | 1 | 14.85 | -2.61 |
| Eurasian | 1 | -36.36 | -40.91 |
| Circumpolar | 14 | -13.49 ±4.42 | -22.84 ±4.93 |
