# Supplementary material for: Projected Range Contractions of European Protected Oceanic Montane Plant Communities: Focus on Climate Change Impacts Is Essential for Their Future Conservation
Source: PLoS One. 2014 Apr 21;9(4):e95147. doi: 10.1371/journal.pone.0095147 (PMC3994024; doi:10.1371/journal.pone.0095147)
Supplement: Table S7 — Outputs from gap analysis, showing the proportional overlap of both current and future distribution of all species with protected areas, using a range of threshold values (≥2%, ≥5%, ≥10%, ≥20%, ≥30%, ≥50% of grid cell occupied by protected areas). (DOC) [file pone.0095147.s007.doc]

Table S7: Outputs from gap analysis, showing the proportional overlap of both current and future distribution of all species with protected areas, using a range of threshold values (≥2%, ≥5%, ≥10%, ≥20%, ≥30%, ≥50% of grid cell occupied by protected areas).

|  | | Current distribution: | | | | | |  | Future distribution: | | | | | |
| --- | --- | --- | --- | --- | --- | --- | --- | --- | --- | --- | --- | --- | --- | --- |
|  | Threshold: | 2% | 5% | 10% | 20% | 30% | 50% |  | 2% | 5% | 10% | 20% | 30% | 50% |
| *Anastrepta orcadensis* | | 1.00 | 1.00 | 1.00 | 0.88 | 0.85 | 0.77 |  | 0.90 | 0.87 | 0.80 | 0.67 | 0.58 | 0.49 |
| *Andreaea alpina* | | 0.91 | 0.91 | 0.85 | 0.67 | 0.60 | 0.54 |  | 0.82 | 0.75 | 0.70 | 0.59 | 0.50 | 0.41 |
| *Anthelia julacea* | | 0.97 | 0.92 | 0.92 | 0.76 | 0.71 | 0.64 |  | 0.86 | 0.84 | 0.80 | 0.69 | 0.58 | 0.49 |
| *Asplenium viride* | | 0.88 | 0.84 | 0.77 | 0.54 | 0.48 | 0.40 |  | 0.77 | 0.69 | 0.62 | 0.47 | 0.40 | 0.32 |
| *Bazzania pearsonii* | | 1.00 | 1.00 | 1.00 | 0.94 | 0.88 | 0.84 |  | 0.95 | 0.94 | 0.91 | 0.83 | 0.75 | 0.61 |
| *Bazzania tricrenata* | | 0.86 | 0.83 | 0.80 | 0.69 | 0.63 | 0.51 |  | 0.81 | 0.77 | 0.71 | 0.57 | 0.49 | 0.39 |
| *Campylopus setifolius* | | 0.94 | 0.87 | 0.81 | 0.72 | 0.69 | 0.56 |  | 0.86 | 0.80 | 0.75 | 0.61 | 0.51 | 0.42 |
| *Carex bigelowii* | | 0.93 | 0.93 | 0.91 | 0.74 | 0.65 | 0.52 |  | 0.83 | 0.78 | 0.74 | 0.61 | 0.52 | 0.43 |
| *Diphasiastrum alpinum* | | 0.81 | 0.74 | 0.70 | 0.55 | 0.49 | 0.39 |  | 0.73 | 0.65 | 0.61 | 0.48 | 0.42 | 0.34 |
| *Empetrum nigrum* | | 0.74 | 0.65 | 0.55 | 0.41 | 0.36 | 0.27 |  | 0.63 | 0.53 | 0.45 | 0.31 | 0.26 | 0.21 |
| *Herbertus aduncus* | | 0.93 | 0.88 | 0.86 | 0.77 | 0.70 | 0.57 |  | 0.87 | 0.82 | 0.77 | 0.63 | 0.54 | 0.43 |
| *Huperzia selago* | | 0.68 | 0.59 | 0.50 | 0.37 | 0.32 | 0.25 |  | 0.64 | 0.54 | 0.47 | 0.35 | 0.29 | 0.23 |
| *Juncus squarrosus* | | 0.61 | 0.46 | 0.38 | 0.28 | 0.23 | 0.18 |  | 0.64 | 0.49 | 0.41 | 0.30 | 0.25 | 0.20 |
| *Mastigophora woodsii* | | 1.00 | 1.00 | 1.00 | 1.00 | 0.94 | 0.89 |  | 0.98 | 0.98 | 0.95 | 0.91 | 0.81 | 0.70 |
| *Mylia taylorii* | | 0.75 | 0.66 | 0.60 | 0.44 | 0.38 | 0.30 |  | 0.68 | 0.59 | 0.53 | 0.40 | 0.33 | 0.26 |
| *Oxyria digyna* | | 1.00 | 0.96 | 0.96 | 0.92 | 0.88 | 0.71 |  | 0.86 | 0.80 | 0.76 | 0.60 | 0.53 | 0.41 |
| *Pleurozia purpurea* | | 0.77 | 0.68 | 0.61 | 0.46 | 0.40 | 0.33 |  | 0.74 | 0.63 | 0.53 | 0.42 | 0.35 | 0.27 |
| *Polystichum lonchitis* | | 0.95 | 0.95 | 0.85 | 0.76 | 0.72 | 0.63 |  | 0.87 | 0.81 | 0.76 | 0.64 | 0.54 | 0.43 |
| *Polytrichum alpinum* | | 0.87 | 0.78 | 0.74 | 0.59 | 0.54 | 0.43 |  | 0.78 | 0.71 | 0.66 | 0.53 | 0.46 | 0.37 |
| *Racomitrium lanuginosum* | | 0.72 | 0.61 | 0.55 | 0.41 | 0.35 | 0.29 |  | 0.67 | 0.56 | 0.50 | 0.38 | 0.32 | 0.25 |
| *Salix herbacea* | | 0.89 | 0.85 | 0.82 | 0.64 | 0.58 | 0.50 |  | 0.89 | 0.85 | 0.82 | 0.71 | 0.62 | 0.49 |
| *Saussurea alpina* | | 1.00 | 1.00 | 0.95 | 0.78 | 0.78 | 0.69 |  | 0.91 | 0.89 | 0.89 | 0.80 | 0.72 | 0.60 |
| *Saxifraga oppositifolia* | | 0.95 | 0.95 | 0.95 | 0.80 | 0.73 | 0.62 |  | 0.84 | 0.81 | 0.77 | 0.66 | 0.57 | 0.46 |
| *Saxifraga stellaris* | | 0.93 | 0.93 | 0.90 | 0.80 | 0.72 | 0.59 |  | 0.83 | 0.77 | 0.72 | 0.55 | 0.46 | 0.38 |
| *Scapania gracilis* | | 0.78 | 0.67 | 0.63 | 0.46 | 0.38 | 0.31 |  | 0.74 | 0.63 | 0.54 | 0.41 | 0.34 | 0.26 |
| *Scapania ornithopodioides* | | 0.94 | 0.94 | 0.94 | 0.85 | 0.85 | 0.81 |  | 0.95 | 0.94 | 0.89 | 0.83 | 0.73 | 0.63 |
| *Sedum rosea* | | 0.92 | 0.89 | 0.84 | 0.71 | 0.62 | 0.49 |  | 0.92 | 0.89 | 0.87 | 0.75 | 0.66 | 0.54 |
| *Thalictrum alpinum* | | 1.00 | 0.96 | 0.96 | 0.81 | 0.68 | 0.59 |  | 0.91 | 0.85 | 0.80 | 0.71 | 0.57 | 0.47 |
| *Vaccinium myrtillus* | | 0.54 | 0.37 | 0.30 | 0.21 | 0.17 | 0.14 |  | 0.55 | 0.40 | 0.32 | 0.24 | 0.20 | 0.16 |
| *Vaccinium vitis-idaea* | | 0.59 | 0.50 | 0.47 | 0.34 | 0.30 | 0.24 |  | 0.36 | 0.27 | 0.26 | 0.19 | 0.15 | 0.11 |
